# Numerical Investigation of the Fracture Mechanism of Defective Graphene Sheets

**DOI:** 10.3390/ma10020164

**Published:** 2017-02-11

**Authors:** Na Fan, Zhenzhou Ren, Guangyin Jing, Jian Guo, Bei Peng, Hai Jiang

**Affiliations:** 1 School of Mechatronics Engineering, University of Electronic Science and Technology of China, Chengdu 611731, China; na_fan@uestc.edu.cn (N.F.); zhenzhouren@gmail.com (Z.R.); jguo@xtu.edu.cn (J.G.); 2 National Key Laboratory and Incubation Base of Photoelectric Technology and Functional Materials, School of Physics, Northwest University, Xi’an 710069, China; jing@nwu.edu.cn; 3 School of Materials Science and Engineering, Xiangtan University, Xiangtan 411105, China; 4 Center for Robotics, University of Electronic Science and Technology of China, Chengdu 611731, China

**Keywords:** graphene, defect, dynamic fracture, finite element method, stress concentration

## Abstract

Despite the unique occurrences of structural defects in graphene synthesis, the fracture mechanism of a defective graphene sheet has not been fully understood due to the complexities of the defects. In this study, the fracture mechanism of the monolayer graphene with four common types of defects (single vacancy defect, divacancy defect, Stone–Wales defect and line vacancy defect) were investigated systematically for mechanical loading along armchair and zigzag directions, by using the finite element method. The results demonstrated that all four types of defects could cause significant fracture strength loss in graphene sheet compared with the pristine one. In addition, the results revealed that the stress concentration occurred at the carbon–carbon bonds along the same direction as the displacement loading due to the deficiency or twist of carbon–carbon bonds, resulting in the breaking of the initial crack point in the graphene sheet. The fracture of the graphene sheet was developed following the direction of the breaking of carbon–carbon bonds, which was opposite to that of the displacement loading.

## 1. Introduction

Graphene has attracted extensive interests in recent years since its experimental discovery in 2004 [1]. Its two-dimensional (2D) hexagonal monolayer network of carbon atoms exhibits extraordinary mechanical, electrical and thermal properties [2], allowing broad applications in a variety of areas such as electrodes, chemical nanosensors, nanocomposites and nanooscillators [3]. To date, a number of methods have been developed for the synthesis of graphene, including chemical vapor deposition (CVD) [4], mechanical exfoliation [5] and ion implantation [6]. However, it has been proven that the structural defects are unavoidable during the growth process regardless of the synthesis method [7,8]. Vacancy defects are defined as the absence of carbon atoms on the graphene sheet, including single and multiple vacancies. Stone–Wales (SW) defect is the nonhexagonal rings formed by reconstruction of graphenic lattice. These structural defects are inevitably introduced during the synthesis or functionalization process of graphene, and may affect the mechanical properties of graphene dramatically [9] by lowering the fracture toughness of the graphene sheets [10]. Therefore, the fracture mechanical behaviors of graphene sheets, in particular the dynamic fracture, are extremely important for the development of graphene-based toughening nano-composites.

Computational simulation is a powerful tool and has been widely used in the study of the fracture behaviors of graphene sheets due to the difficulties of precise defect control during the experimental measurements. For example, Zhang et al. [11] revealed the nanofracture in graphene sheet under complex mechanical stresses using molecular dynamics (MD) simulation. Wang et al. [12] studied the mechanical properties of the graphene with a family of 5-8-5 defects by using MD simulation. Xu et al. [13] proposed a coupled quantum/continuum mechanics approach to study the crack propagation from the edge of the graphene sheet. Georgantzinos et al. [14] reported a nonlinear structural mechanics approach examined the impact of pinhole defects on the fracture of graphene. He et al. [15] investigated the effects of the orientation and tilting angles of the Stone-Thrower-Wales (STW, 5-7-7-5) defects based on MD simulation. They found that the breaking strength of graphene decreased with the increasing tilting angle. However, the MD simulations are limited in both length scales and the time consumption. Alternatively, finite element (FE) models based on continuum theory have been adopted to overcome the weakness in the MD simulations. For instance, Xiao et al. [16] studied the fracture and progressive failure of a defective (SW defect) graphene sheet by FE simulation. Baykasoglu et al. [17] developed an atomistic based finite element model for predicting the fracture in both SW and single vacancy defective graphene sheets. Their results showed that the graphene sheets exhibited an orthotropic fracture behavior. Canadija et al. [18] studied the effect of vacancy location and the density of vacancies on the bending behavior of graphene sheets by using a FE-based structural mechanics approach. Recently, Zhang et al. [19] used FE method to predict the instability of dynamic fracture in a line defective graphene sheet.

As mentioned above, many efforts have been made to investigate the fracture of the defective graphene sheets, however, a comprehensive and comparative study of mechanical properties contributed from all the typical defects have not been systematically studied, especially the process of the dynamic fracture of graphene sheet. In this study, the FE model based on molecular structural mechanics was established with the geometric nonlinear effects taken into account, to study the mechanical properties and dynamic fracture of graphene sheet with four different types of defect (single vacancy (SV) defect, divacancy (DV) defect, SW defect and line vacancy (LV) defect). This numerical study revealed the fracture mechanism of these four types of defective graphene sheets, which could serve as a benchmark to search for an effective way for inhibiting the fracture of the graphene sheets.

## 2. Model and Methodology

### 2.1. Equivalent Nonlinear Timoshenko Beams for Covalent Carbon–Carbon (C–C) Bonds

In order to simulate the fracture of graphene, an equivalent model of graphene must be established. The large deformation and nonlinear geometric effects shall not be ignored in the fracture analysis process. Considering the realistic distributions of thickness and equilibrium length of the C–C bonds, the covalent bond could be equated to nonlinear Timoshenko beams in this study, as presented by the linear planar beam elements (Beam 21) in Abaqus 6.10. The constants of the beam elements were computed and listed in Table 1 [19,20]. Belytschko et al. [21] noted that once the length of a C–C bond exceeded the cut-off distance *r*_c_, the interatomic force would drop rapidly to zero, and the bond was regarded as broken. The interaction range of carbon atoms was confined within a distance *r*_c_ = 0.17 nm to avoid unphysical spurious bond forces [21]. The dynamic fracture process would be shown by the successional breakages of C–C bonds. Besides, the accurate nonlinear constitutive relation of the equivalent beam for a C–C bond could be revised as follow [19]:

(1) $$\sigma =20.852\left(-e^{-4.658\varepsilon }+e^{-3.817\varepsilon }\right)$$

where σ is the stress of the C–C bond, and ε is the strain of the C–C bond. According to the stress–strain curve of the equivalent beam, the critical strain ε_c_ and critical stress σ_c_ were 0.197 and 1501 Gpa, respectively. The C–C bond broke when it reached the critical stress σ_c_. The material constitutive relation of beam (Equation (1)) was coded as VUMAT subroutine in ABAQUS/Explicit.

### 2.2. FE Model of the Pristine Graphene and Verifications

2D FE models were constructed by Beam21 (shear) and Mass elements in ABAQUS. In the model of graphene, a carbon atom was represented by a node at either end of the beam element, while the beam reflected the reaction between nodes. The dynamic fracture process would be shown by the following breaking of C–C bonds.

**Figure 1:**
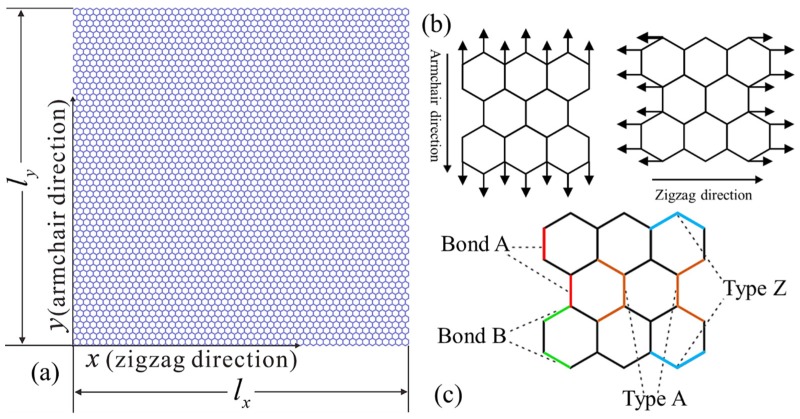
(**a**) The equivalent FE model of the pristine graphene sheet. (**b**) Two directions of the displacement loading, AC direction and ZZ direction. (**c**) Schematics of the configurations of the C–C bonds.

Figure 1a illustrates the equivalent FE model of the pristine graphene sheet. The dimensions of the defect-free model of graphene were 12.5520 nm (*l_x_*) × 12.6470 nm (*l_y_*). It contained 6180 carbon atoms (nodes) and 9159 bonds (elements). To obtain the mechanical properties and dynamic fracture of the graphene model, uniform normal loadings of displacement were applied to nodes on one edge, as shown in Figure 1. In order to facilitate the subsequent analysis, we defined the configurations of the C–C bonds as shown in Figure 1c. Bond A denoted that the C–C bond paralleled to the armchair direction (AC direction). Bond B denoted that the C–C bond intersected the zigzag direction (ZZ direction) with a 30° angle. The armchair structural configuration consisting of one Bond A and two Bond B was denoted as Type A, while the zigzag structural configuration consisting of two Bond B was denoted as Type Z.

**Figure 2:**
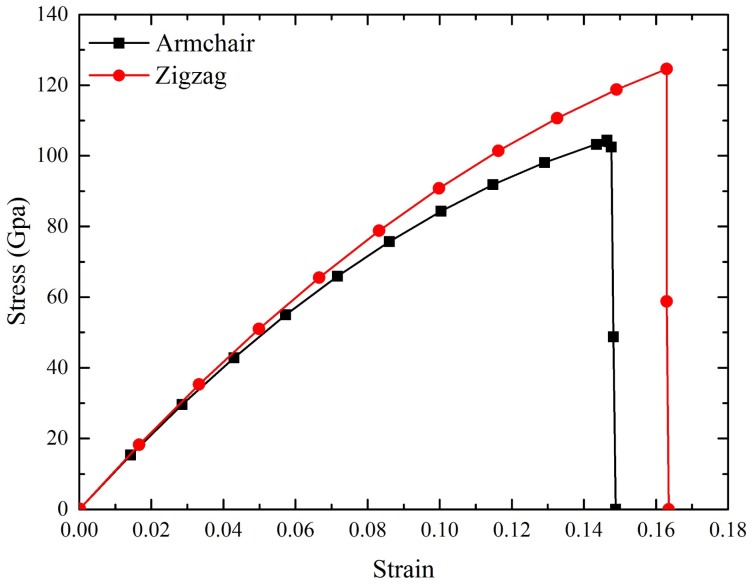
Stress–strain curves of defect-free graphene.

To validate the FE model, the mechanical properties of a pristine graphene was firstly analyzed by using the loading parameters. All of the models were applied a uniaxial displacement loading along AC and ZZ direction, respectively (Figure 1). The loading time was longer than the minimum natural period (the natural period could be obtained through the simulation) due to the quasi-static fracture process of graphene. Therefore, the loading time was at least 6.75 ps while the loading time must also be sufficient to ensure the occurrence of fracture. In the study, the strain rate ε was set at ε = *Δl*/(*l·t*) = 9.84 × 10^–4^ fs^–1^ with a loading time of 18 ps. Along AC direction (*l* = *l_y_* = 12.6470 nm), the displacement loading *Δl* on the both ends of the model was 1.1200 nm. At the ZZ direction (*l* = *l_x_* = 12.5520 nm), the displacement loading *Δl* was 1.1116 nm. Figure 2 showed the calculated stress–strain curves of the pristine graphene under the loading along AC and ZZ directions, respectively. The fracture stress (σ_f_) and fracture strain (ε_f_) along the AC and ZZ directions were 104.1 GPa, 122.8 GPa and 0.149, 0.164, respectively. Both of the curves fell within the previous simulation study [17,22,23,24], i.e., σ_f_: 90–125 GPa (AC direction), 110–125 GPa (ZZ direction) and ε_f_: 0.14–0.21 GPa (AC direction), 0.14–0.24 GPa (ZZ direction). The Young’s modulus *E* and Poisson’s ratios μ of the pristine graphene sheet could be calculated as follow:

(2) $$\text{AC direction}\;E=\frac{mF_{p}l_{y}}{tl_{x}\Delta l_{y}},\;\mu =-\frac{\varepsilon_{lx}}{\varepsilon_{ly}}$$

(3) $$\text{ZZ direction}\;E=\frac{mF_{p}l_{x}}{tl_{y}\Delta l_{x}},\;\mu =-\frac{\varepsilon_{ly}}{\varepsilon_{lx}}$$

where *m* is number of loading points (120 for AC direction and 103 for ZZ direction), *F*_p_ is the force of the single loading point and *t* is the thickness of the model (0.334 nm). The obtained Young’s modulus and Poisson’s ratios of the pristine graphene sheet also corresponded closely to the experimental values (Young’s modulus of *E* = 1.0 ± 0.1 TPa [25]) as shown in Table 2.

### 2.3. FE Model of the Defective Graphene

**Figure 3:**
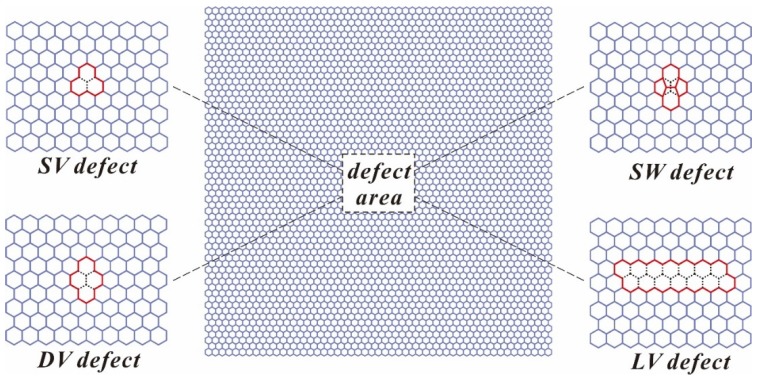
Equivalent FE model of the defective graphene (the dash line indicates the missing or rotating C–C bonds).

The structural defects were generated inevitably during the production or chemical functionalization process of graphene. In this study, we investigated four typical types of defects (SV defect, DV defect, SW defect and LV defect) in the graphene sheet, as shown in Figure 3. In all models, the defect was located at the center of the graphene sheet. The SV defect was formed by missing one carbon atom and three C–C bonds (one Bond A and two Bond B), and the DV defect was formed by missing two carbon atoms and five C–C bonds (one Bond A and four Bond B). The SW defect was formed by the result of 90° rotation of the central C–C bond, and the LV defect was formed by a line of DV defect (12 Bond A and 13 Bond B) along the ZZ direction.

## 3. Results and Discussion

### 3.1. Dynamic Fracture of Graphene with Different Defects

**Figure 4:**
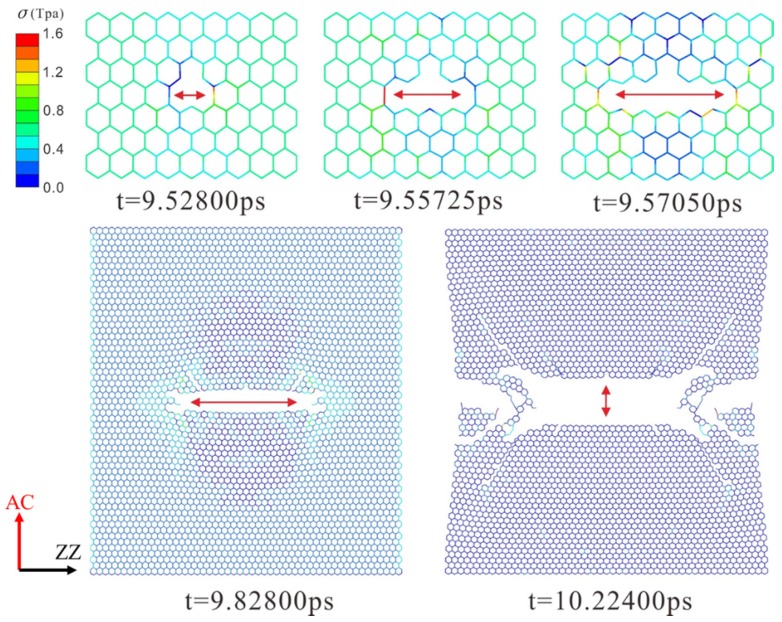
The crack propagation of the SV defect under loading along AC direction.

**Figure 5:**
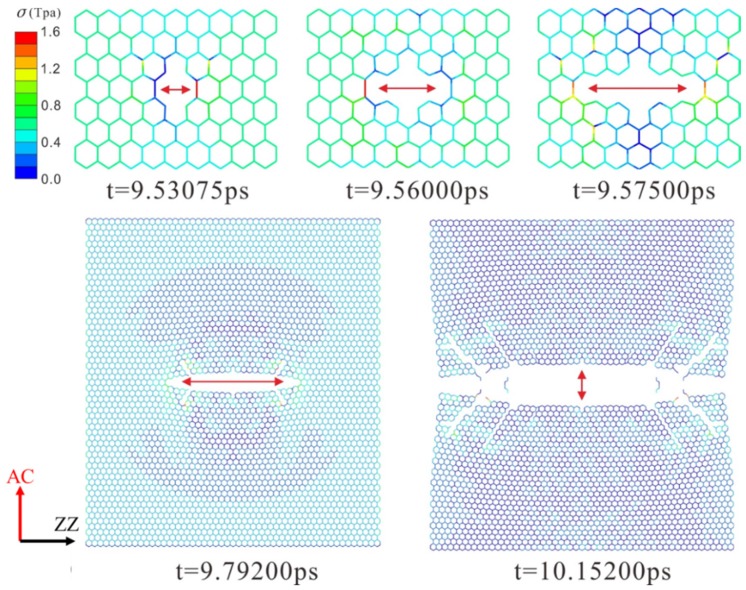
The crack propagation of the DV defect under loading along AC direction.

**Figure 6:**
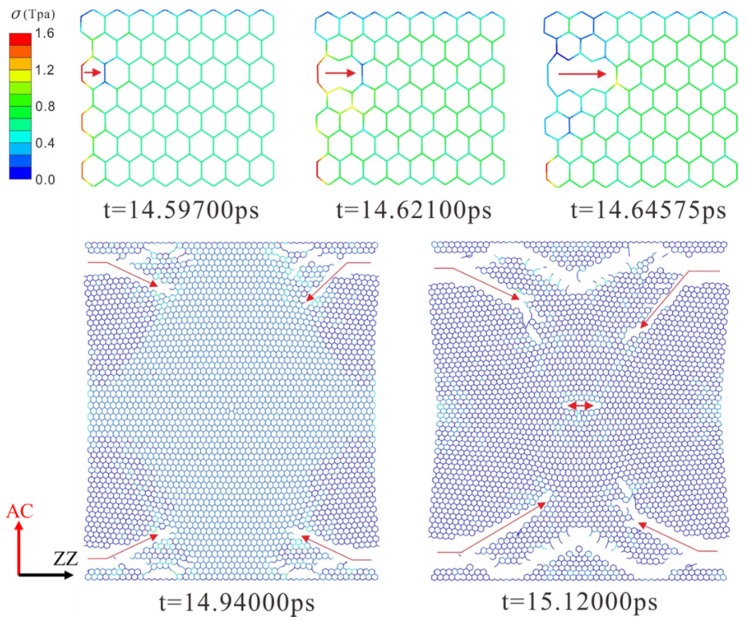
The crack propagation of the SW defect under loading along AC direction.

**Figure 7:**
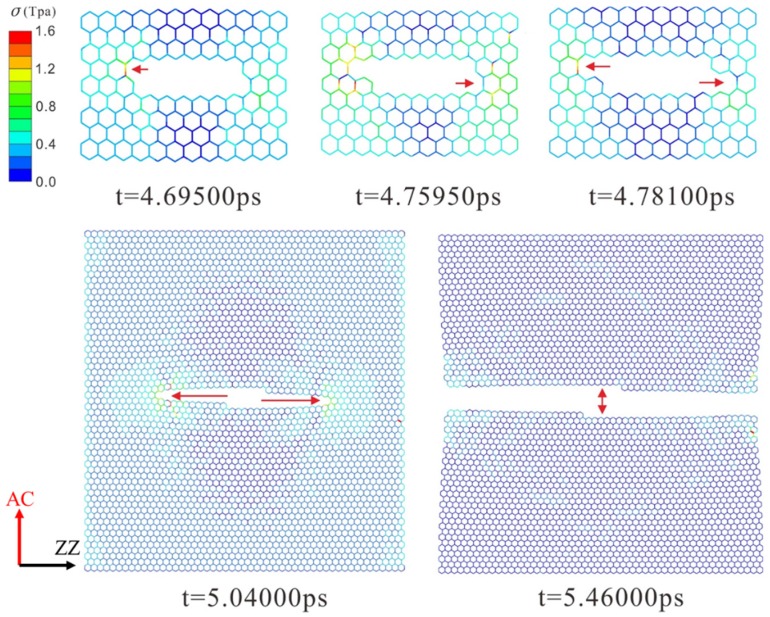
The crack propagation of the LV defect under loading along AC direction.

**Figure 8:**
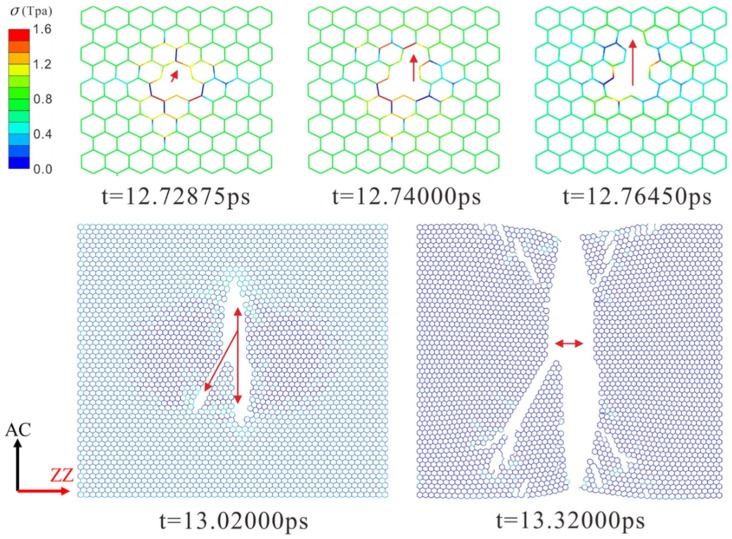
The crack propagation of SV defect under loading along ZZ direction.

**Figure 9:**
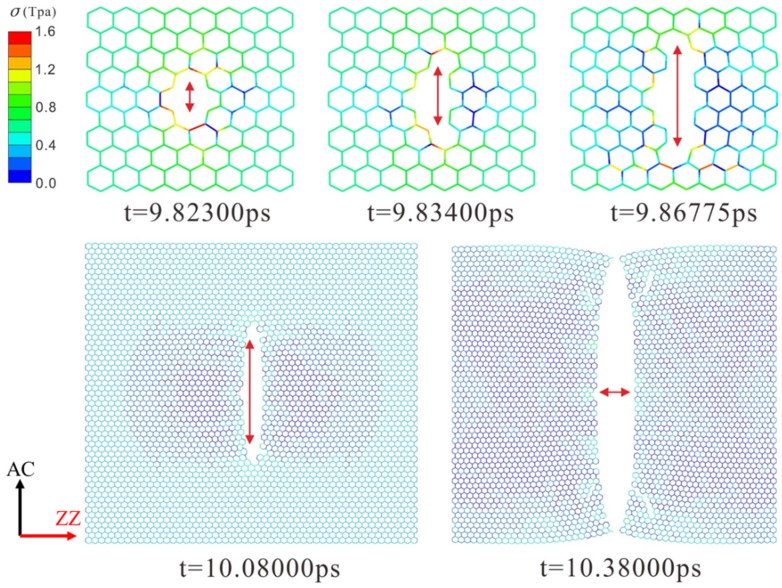
The crack propagation of DV defect under loading along ZZ direction.

**Figure 10:**
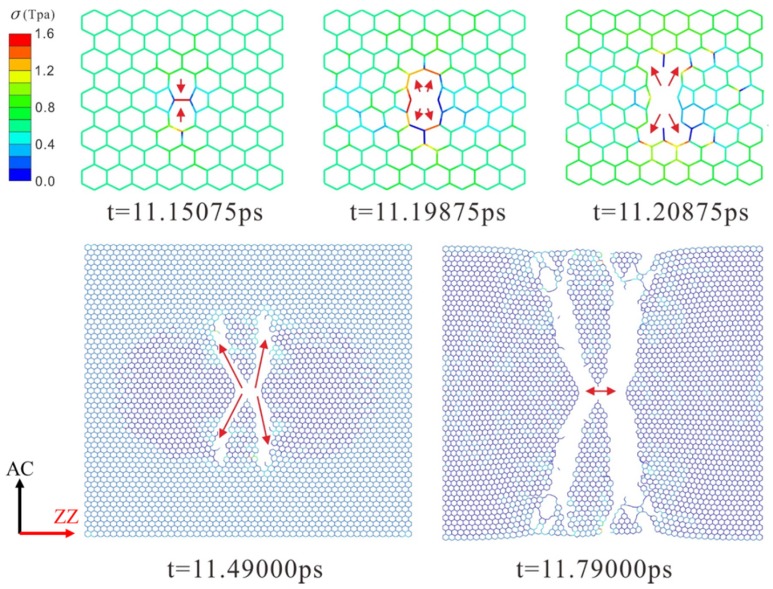
The crack propagation of SW defect under loading along ZZ direction.

**Figure 11:**
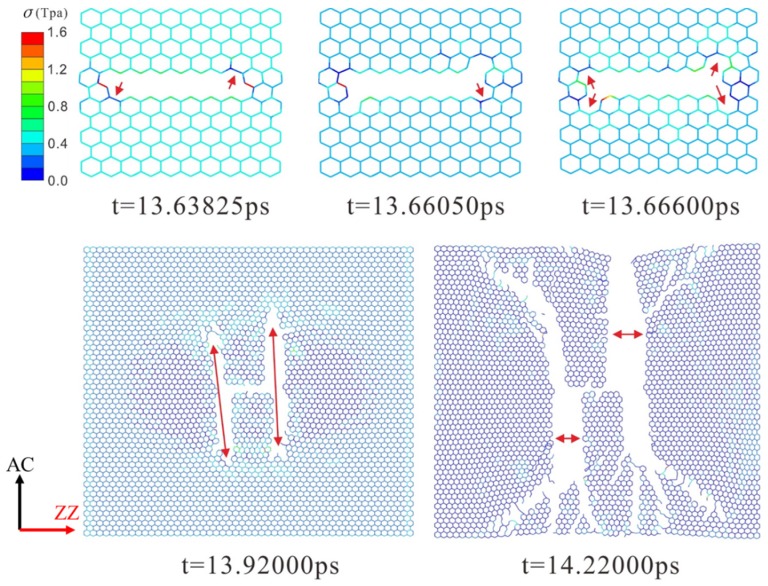
The crack propagation of LV defect under loading along ZZ direction.

The dynamic fracture and propagation in graphene by FE analysis with the four different types of defects under loading stress along AC and ZZ directions are shown in Figure 4, Figure 5, Figure 6 and Figure 7 (Supplementary Videos 1–4) and Figure 8, Figure 9, Figure 10 and Figure 11 (Supplementary Videos 5–8), respectively. As the displacements of both ends of the graphene increased gradually, the C–C bond broke when it reached the critical stress σ_c_ = 1501 GPa, resulting in the crack propagation instantly. As could be seen in these figures, the propagation of the crack presented was radically symmetrical due to the geometric symmetry of the model. The occurrence of the branches was observed during the propagation of crack, which agreed with the previous studies by Omeltchenko [26] and Zhang [27]. From all the fracture analysis, we found that the cracks were always initiated from the defective area, therefore all these four typical types of defects were considered had various influence on the dynamic fracture of graphene.

It was obvious that the deficiency of the C–C bonds occurred in the SV defect, DV defect and LV defect. When applying loading stress along the AC direction as shown in Figure 4, Figure 5 and Figure 7, the stress concentration generated around the defective area but only on the Bond A of the Type A (Figure 1c). For example, in Figure 4, there were two symmetrical Type A, the stress concentration occurred on the two Bond A of the Type A. The breaking firstly occurred on the two Bond A due to the stress concentration, which we called it “initial crack point”. With the time, the crack was propagating until the fracture of the whole graphene sheet, resulting in the crack propagating to the ZZ direction in a line. This phenomenon also was found in Figure 5 and Figure 7. The crack propagation direction of SW defective graphene (Figure 6) was different from those with the vacancy defects since there was no missing C–C bond. It was shown in Figure 6 that the stress bearing area was at the edge of the model rather than the defective site. Hence, the initial crack point of the SW defect was not at the defect site but at the corners of the graphene sheet. This was because the C–C bond was hard to break under the loading force along the AC direction due to the complete C–C bonds. The stress concentration appeared at the corners.

Under the loading stress along the ZZ direction (as shown in Figure 8, Figure 9, Figure 10 and Figure 11), due to the missing Bond B in the SV defect, DV defect, and LV defect, the local stress concentration could generate at Bond B around the defective area. As shown in Figure 8, local stress concentration occurred at the Bond B in the Type Z. Since there was only one Type Z on the top but two at the bottom, the stress concentration initially generated on the top and then the bottom, followed by the occurrence of a Y-shaped crack. In the DV defective graphene (Figure 9), the stress concentration would generate at Bond B at the both of the top and bottom Type Z and cause two initial crack points. The crack propagation was along the armchair direction and formed an I-shaped crack. As shown in Figure 11, in the LV defective graphene sheet, the stress concentration was found occurred at the four Bond B in Type Z thus four initial cracks and an H-shaped crack were formed. In the SW defective graphene, as shown in Figure 10, although there was no lack of the C–C bond, there was one C–C bond that had the same direction as the loading stress due to the rotation of the C–C bonds. This C–C bond could easily break caused by the stress concentration under the loading stress along the ZZ direction. Afterwards, four initial crack points generated at the four Bond B at both the top and bottom defective area, resulting in an X-shaped crack.

### 3.2. Fracture Strength of Graphene with Different Defects

**Figure 12:**
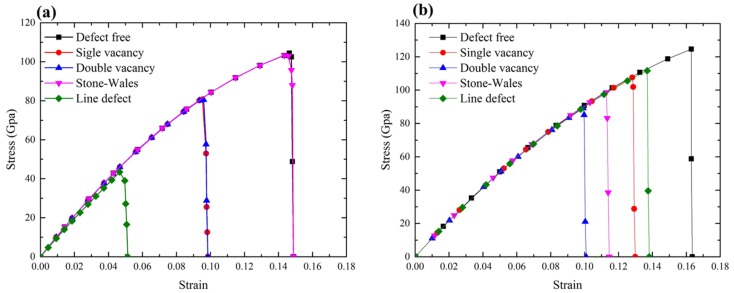
Stress–strain curves of the pristine and defective graphene under loading along: (**a**) AC direction; and (**b**) ZZ direction.

In order to give a comprehensive and comparison studies of mechanical properties of all the typical defects, the fracture strength of graphene with those four defects has been studied. The stress–strain curves of these defective graphene under loading were calculated along both the AC and ZZ directions, as shown in Figure 12. As a comparison, the stress–strain curve of the pristine graphene was also included in the plots.

Figure 12a demonstrated the effect of these defects on the fracture strength of graphene along the AC loading direction by comparing that of the pristine graphene. The fracture stress of the pristine graphene was 104.1 GPa under the loading along AC direction, as shown in Figure 12a, while it was only 43.2 GPa for the graphene with LV defect, which was decreased by 60.4%. In this case, the stress concentration occurred on the two Bond A of the Type A located at the edge of the defective area as shown in Figure 7, the cracks of these two rows propagated coinstantaneous in opposite directions. This result showed that the line defect had a substantial effect on the fracture strength of graphene along the AC direction.

It was found that SW defect had very little impact on the fracture strength of graphene under the loading along AC direction. The fracture stress of graphene sheet with SW defect was 103.7 GPa, which was only 0.3% less than that of the defect-free graphene sheet. Because the Bond A did not miss in this case, no stress concentration occurred on the defective area along the AC loading direction. The initial crack point of the SW defect was not at the defect site but the corners of the graphene sheet. This also could be used to explain that why the fracture stress in SW defect caused by AC loading was similar to that in the pristine graphene.

In addition, the fracture stress of graphene with SV defect and DV defect were almost identical, 80.0 GPa and 79.6 GPa, which were decreased by 23.2% and 23.5%, respectively. As shown in Figure 4 and Figure 5, both of them missed the structure, one Bond A, the stress concentration occurred at the same sites, thus they had the closed locations of the initial crack points and propagation as well as the fracture stress.

However, under the loading along ZZ direction, as shown in Figure 12b, the LV defect made the fracture stress of graphene only decrease by 10.5% to 105.4 GPa compared to 122.8 GPa of the pristine graphene, which was quite different to the decrease of 60.4% of the fracture stress in AC loading direction. The LV defect missed 12 Bond A and 13 Bond B, however there were seven Type Z (14 Bond B) located at the top and bottom of the defective area. This might reduce the stress concentration at Type Z because these Bond B would share the loading stress evenly and it needed more loading stress to generate the stress concentration at the left and right of the edge of the defective area. However, along AC direction, only one Type A individually exists at the top and bottom of the defective area, it is more likely to have local stress concentration thus resulting in the fracture. The DV defect made the fracture stress of graphene sheet decrease by 29.8% to 86.2 GPa and the SV defect made it decrease by 14.2% to 109.9 GPa, respectively. This was because the SV defect had one more Type Z to share the loading stress compared with the DV defect, which meant that the SV defect needed more loading stress to generate the stress concentration. The SW defect made the fracture stress decrease by 21.2% to 96.8 GPa. These are radically different from the decrease effects on the AC direction (decrease of 0.3%). This was because once applied the loading along the ZZ direction, the stress concentration was generated on one C–C bond (Figure 10) at the defective area.

## 4. Conclusions

In this study, the molecular structural mechanics based finite element models of monolayer graphene with SV defect, DV defect, SW defect and LV defect were developed to study the fracture strength and dynamic fracture, thus revealing the mechanism of fracture failure of the defective graphene. The numerical simulation results demonstrated that the missing and rotation of the C–C bonds may lead to a stress concentration at the defective area along the same direction as the displacement loading, resulting in the breaking of the covalent C–C bonds, and generating the initial crack points. The crack propagated along the crack of the C–C bonds and caused the fracture of the graphene sheet. The breaking of the C–C bonds by the stress concentration significantly reduced the fracture strength of the graphene. The LV defect had the largest effect on the fracture strength of graphene under loading along the AC direction which the DV defect presented the largest effect along the ZZ direction.

Overall, the fracture of defective graphene would be attributed to the stress concentration caused by the breaking of C–C bonds and the initial crack point at the defective site along the displacement loading direction. An effective solution to control the fracture is to decrease the stress concentration in the defective area, for example, layer stack to stagger the defective area. Zhi-Min Liao et al. [28] found that the fracture force distribution of the stacked graphene was very different from that of monolayer graphene. The membrane of stacked graphene became less sensitive to the defects during nanoindentation. Stacked graphene may reduce the stress concentration compared with the monolayer graphene. All the results in the present study provide researchers more valuable information, thus helping to prevent unexpected catastrophic failure in various graphene applications.

## Figures and Tables

**Table 1:** Constants of the beam elements [19,20].

Bond cross-sectional diameter, *d*	0.089 nm
Bond length, *r*_0_	0.142 nm
Cross-sectional area, *A*	6.22 × 10 ^–3^ nm^2^
Moment of inertia, *I*_b_	3.08 × 10 ^–3^ nm^4^
Young’s modulus, *E*_b_	19.5 TPa
Poisson’s ratio, μ_b_	0.23
Shear modulus, *G*_b_	7.93 TPa

**Table 2:** Young’s modulus and Poisson’s ratios of pristine graphene sheet

Direction	Young’s modulus (TPa)	Poisson’s Ratio
AC direction	1.075	0.172
ZZ direction	1.096	0.162

## Supplementary Materials

The following are available online at www.mdpi.com/1996-1944/10/2/164/s1, Videos 1–8.
